# Estimation of baboon daily travel distances by means of point sampling – the magnitude of underestimation

**DOI:** 10.5194/pb-4-143-2017

**Published:** 2017-07-10

**Authors:** Holger Sennhenn-Reulen, Langhalima Diedhiou, Matthias Klapproth, Dietmar Zinner

**Affiliations:** 1 Cognitive Ethology Laboratory, German Primate Center, Leibniz-Institute for Primate Research, Kellnerweg 4, 37077 Göttingen, Germany; 2 Leibniz ScienceCampus “Primate Cognition”, German Primate Center/Leibniz Institute for Primate Research, Kellnerweg 4, 37077 Göttingen, Germany; 3 Direction Parc National du Niokolo-Koba, Tambacounda, Senegal

## Abstract

Daily travel distance (DTD), the distance an animal moves over the course of the day, is an important metric in movement ecology. It provides data with which to test hypotheses related to energetics and behaviour, e.g. impact of group size or food distribution on DTDs. The automated tracking of movements by applying GPS technology has become widely available and easy to implement. However, due to battery duration constraints, it is necessary to select a tracking-time resolution, which inevitably introduces an underestimation of the true underlying path distance. Here we give a quantification of this inherent systematic underestimation of DTDs for a terrestrial primate, the Guinea baboon. We show that sampling protocols with interval lengths from 1 to 120 min underestimate DTDs on average by 7 to 35 %. For longer time intervals (i.e. 60, 90, 120 min), the relative increase of deviation from the “true” trajectory is less pronounced than for shorter intervals. Our study provides first hints on the magnitude of error, which can be applied as a corrective when estimating absolute DTDs in calculations on travelling costs in terrestrial primates.

## 1 Introduction

Spatial information is crucial for many questions in ecological and behavioural research, e.g. species or resource distribution, habitat utilisation and estimates of home ranges or daily travel paths. The application of a satellite-supported global positioning system (GPS) has improved the collection and accuracy of spatial data (Kays et al., 2015), providing ecologists and behavioural biologists with opportunities to determine spatial patterns and test spatially explicit hypotheses. Similarly, the use of GPS has become more prevalent in primate field studies (Osborne and Glew, 2011; Sterling et al., 2013). Beside the determination of geographical positions of ecological objects or structures within a primate's home range – such as sleeping and resting sites, feeding patches or seed-dispersal events – spatial data have been used to estimate home ranges (position, shape and size), habitat utilisation, and daily travel paths and travel distances. In primatology, the application of GPS collars indicated great potential particularly for semi-terrestrial primates in (semi-)open habitats (Markham and Altmann, 2008), but also for arboreal species (Stark et al., 2017).

**Table 1:** A selection of GPS fixing intervals applied in primate and non-primate studies.

Species	Sampling interval	Device	Reference
*Papio anubis*	“Continuously” at 1 Hz	Collar	Strandburg-Peshkin et al. (2015)
*Chlorocebus*	15 min	Collar	Isbell and Bidner (2016)
*Papio ursinus*	20 min	Collar	Hoffman and O'Riain (2012)
*Papio ursinus*	1 h	Collar	Pebsworth et al. (2012a, b)
*Papio cynocephalus*	1 h	Collar	Markham and Altmann (2008)
*Macaca fuscata*	1 h	Collar	Sprague et al. (2004)
*Nasalis concolor*	1 h	Collar	Stark et al. (2017)
*Papio ursinus*	3 h	Collar	Hoffman and O'Riain (2012)
*Rhinopithecus bieti*	2–5 fixes day${}^{-1}$	Collar	Ren et al. (2008)
*Rhinopithecus bieti*	2–5 fixes day${}^{-1}$	Collar	Ren et al. (2009)
*Gorilla beringei*	30 s	Handheld	Wright et al. (2015)
*Papio cynocephalus*	5 min	Handheld	Johnson et al. (2015)
*Papio ursinus*	Average 9 min	Handheld	Bonnell et al. (2015)
*Chiropotes sagulatus*	Average 10 min	Handheld	Gregory et al. (2014)
*Papio ursinus*	20 min	Handheld	Hoffman and O'Riain (2012)
*Macaca silenus*	30 min	Handheld	Santhosh et al. (2015)
*Rhinopithecus bieti*	30 min	Handheld	Grueter et al. (2008)
*Hoolock leuconedys*	30 min	Handheld	Sarma and Kumar (2016)
*Equus caballus*	5 s for 6.5 days	Collar	Hampson et al. (2010)
*Panthera tigris*	1–3 h	Collar	Naha et al. (2016)
*Capra hircus*	2 h	Collar	Chynoweth et al. (2015)
*Elephas maximus*	8 h	Collar	Alfred et al. (2012)
*Canis lupus*	0.25, 1.5, 2, 6, 12 h	Collar	Mills et al. (2006)

Either animals can be equipped with a GPS device, and the respective positions will be collected automatically at pre-programmed intervals, or a researcher follows an animal and determines the positions using a handheld device (e.g. see Table 1). The GPS device consumes energy for every location fix, and thus battery life limits the number of position attempts or fixes a device can do. Programming fewer GPS fixes results in longer battery life but at the price of lower data density. It might not be a big problem if one is interested in the area an animal uses within a year, which one can probably estimate fairly well with just 2 or 3 fixes day${}^{-1}$ (Cagnacci et al., 2010). However, it can be problematic if one is interested in daily travel distance (DTD), where, optimally, a quasi-continuous recording of the travel path would be best, e.g. 1 fix s${}^{-1}$ (1 Hz). In many studies, a trade-off between long battery life for collecting data over a longer time period to estimate annual home ranges and a high data density to estimate DTD is sought. In particular the number of fixes per day used to estimate DTDs can influence the accuracy of the estimates and can make comparative studies within and between species difficult (e.g. Johnson et al., 2015).

Uncertainties in animal movement data, owing e.g. to sampling frequency, may strongly influence interpretations of tracking data (Bradshaw et al., 2007; Harris and Blackwell, 2013; Laube and Purves, 2011). As expected, in a number of studies it was shown that, as sampling intervals increase, the uncertainty of the behaviour between fixes increases; e.g. DTDs estimated from low sampling frequencies were significantly shorter than those based on higher sampling frequencies (Laundré et al., 1987; Mills et al., 2006; Reynolds and Laundré, 1990; Rowcliffe et al., 2012; Edelhoff et al., 2016). How, if at all, this effect can be corrected statistically or by modelling is an open question (Blackwell et al., 2016; Fleming et al., 2014a, b, 2016; Shamoun-Baranes et al., 2011). One way to mitigate these effects can be an empirical estimation of the magnitude of error one yields by applying different sampling frequencies.

**Figure 1:**
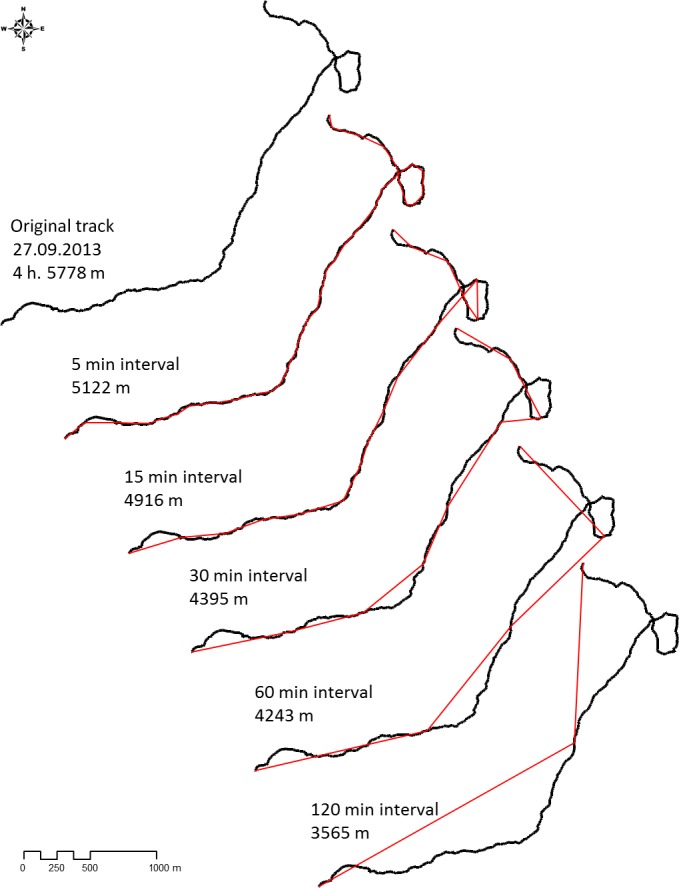
Example of a baboon track line (black) over 4 h and estimated travel distances if sampling is done applying different interval lengths (respective red lines).

In a study on range use of Guinea baboons (*Papio papio*) in the Niokolo-Koba National Park (Parc National du Niokolo-Koba, PNNK), Senegal, we equipped baboons with Tellus ultra-light GPS remote ultra-high-frequency (UHF) collars (Televilt, TVP Positioning AB, Lindesberg, Sweden; nowadays Followit AB). Since the main purpose of the application of GPS collars was to estimate home ranges of the baboons rather than an analysis of DTDs and since battery longevity was limited, we programmed the collars to take only 10 fix day${}^{-1}$ (seven fixes between 06:00 and 18:00 at 2 h intervals, and three fixes at night at 21:00, 00:00 and 03:00) but over a longer period (on average 10 months). Even with a sampling interval of 2 h and thus just seven location points per day (the night-time fixes were not used since the baboons remained mainly stationary during the night), it was possible to approximate DTDs which could at least be used for inter-individual comparisons within the same population, given that the error in estimating DTDs was similar for all collared baboons. However, absolute DTDs were expected to be much longer than those approximations based on just seven location points (Fig. 1), making estimations on actual travelling costs unreliable and comparisons of DTDs with other studies problematic.

In our study we therefore aimed to estimate the magnitude of error when determining DTDs for Guinea baboons (*Papio papio*) by applying various sampling intervals instead of continuous tracking. For this we compared “true” DTDs with DTDs estimated by using 1, 2, 5, 10, 15, 30, 60, 90 and 120 min sampling intervals. The true DTD derived from direct, quasi-continuous GPS tracking of focal baboons; i.e. a baboon was followed by a researcher carrying a handheld GPS device recording a continuous track. Further, one can expect that if a baboon moves more or less in a straight line the error might be smaller than in cases when the baboons meander a lot (e.g. Postlethwaite and Dennis, 2013). We therefore explored which particular travel behaviours of the baboons resulted in a greater or smaller deviation from the true DTD.

**Table 2:** Temporal distribution of tracking periods. Tracking periods were either 4 or 2 h long. ID: individual baboon males; numbers in first horizontal line indicate hours of the day. T: tracking periods included in analysis; t: tracking periods excluded, because of gaps in the continuous tracking larger than 60 s.

ID	Date (dd.mm.yyyy)	5	6	7	8	9	10	11	12	13	14	15	16	17	18
MST	08.09.2013			T	T	T	T						T	T	
MST	09.09.2013			T	T	t	t						T	T	
SNE	10.09.2013				T	T									
JKY	14.09.2013				T	T	T	T							
OSM	15.09.2013				T	T									
MST	16.09.2013				T	T									
SNE	18.09.2013			T	T	T	T								
JKY	19.09.2013			t	t										
MST	20.09.2013			t	t		T	T					T	T	
SNE	21.09.2013			t	t	T	T								
OSM	23.09.2013			T	T	T	T								
OSM	25.09.2013				T	T	T	T							
JKY	26.09.2013				t	t	T	T							
SNE	27.09.2013			T	T	t	t								
JKY	28.09.2013				T	T	T	T							
OSM	06.10.2013			t	t	T	T								
JKY	07.10.2013			T	T	T	T								
JKY	19.10.2013			T	T	T	T								
MST	20.10.2013			T	T	T	T								
OSM	21.10.2013				T	T							T	T	
SNE	22.10.2013			T	T	T	T								
JKY	23.10.2013			T	T	T	T						T	T	
OSM	24.10.2013			T	T	T	T						T	T	
MST	25.10.2013			t	t	T	T					T	T		
SNE	26.10.2013			t	t	T	T					T	T		
JKY	27.10.2013		T	T	T	T						T	T		

## 2 Methods

### 2.1 Study site and subjects

The study was carried out in the Niokolo-Koba National Park at the research station of the German Primate Center in Simenti (13${}^{\circ }$01${}^{\prime }$34${}^{\prime \prime }$ N, 13${}^{\circ }$17${}^{\prime }$41${}^{\prime \prime }$ W). The habitat consists of a forest–savannah mosaic with seasonally flooded grassland, dry deciduous forest and gallery forest along the Gambia River. The climate is characterised by a dry season from November until May and a rainy season from June until October.

The baboon community in Simenti comprises 350–400 individuals. They live in a multi-level society consisting of one-male units (OMUs), parties and gangs (Patzelt et al., 2014; Goffe et al., 2016). The baboons were habituated to human observers, so that observations and follows could be done from less than 5 m distance.

We selected four males from different parties, and one of us (Langhalima Diedhiou) followed on foot one individual baboon at a time, keeping a distance of 5 m to the respective focal animal. The follows were repeated several times for each male (Table 2). The respective tracks were recorded with a handheld Garmin GPSMAP 62. Tracing set-up was “auto-normal”. In sum we recorded 56 2 h tracks. In nine cases we experienced gaps in the continuous recording of the respective tracks (leg time > 60 s). We deleted these nine tracks from our analyses.

### 2.2 Statistical analysis

#### 2.2.1 Deviation from true travel distance

Using a GPS device, even a continuous track consists of a number of fixes, optimally with a very short sampling interval or leg time. Leg time is the delta between the time stamps of the two fixes bounding the leg (e.g. 1 s if the sampling frequency is 1 Hz). However, since conditions are not always optimal, the real leg time varies and is most often larger than the targeted 1 s leg time. As a result, when we overlaid the continuous track with a 1 min sampling interval, for instance, the respective 1 min time stamps did not necessarily match with a fix from the GPS device. For instance, the closest time stamps can be at 57 or 62 s instead of 60 s. Therefore we had to interpolate the tracks and re-discretise them.

We artificially re-discretised the original tracks with regular sampling intervals of 1, 2, 5, 10, 15, 30, 60, 90 and 120 min (shown on the x axis of Figs. 3 and 4) by using linear interpolation between coordinates from the original tracks where necessary. This was achieved using the function “redisltraj” in the “adehabitatLT” R package (Calenge, 2006). The deviation from the original travel distance is the difference between the original travel distance and the travel distance of the re-discretised version.

To get to a relationship between the deviation from the original travel distance and the re-discretisation sampling interval duration, we fitted a Bayesian multilevel log-normal regression model using the Stan-based (Stan Development Team, 2015) R add-on package brms (Bürkner, 2017), with track index as grouping factor $\gamma_{i}$. The conditional mean of the deviation from the original travel distance y across the re-discretisation sampling interval duration range of t∈[1,120] was modelled as a $\frac{y}{-15}$ transformed response (log-normally distributed, therefore with a $\log_{e}$ link function) on the basis of the linear predictor $\beta_{0}+\beta_{1}\cdot t+\beta_{2}\cdot {\mathrm{log}}_{e}\left(t\right)+\gamma_{i}$, which leads to an improved expected predictive accuracy (based on the leave-one-out information criterion; Vehtari et al., 2016) in comparison to a set of other potential non-linear transformations, including a linear relationship, and power transformations of higher order. Section S1 in the Supplement gives more details on the applied statistical approach, as well as posterior mean and credible interval estimates for $\beta_{0}\beta_{1}\beta_{2}$.

#### 2.2.2 Hidden Markov model

To be able to further quantify how the deviation from the original travel distance is related to moving velocity and turning-angle states, we fitted a hidden Markov model (Michelot et al., 2016). As an example, we performed this for a re-discretisation duration of 5 min, which enables us to classify the underlying moving states on the basis of this coarsened information. This grid is still short enough – and therefore close enough to our original quasi-continuous sampling – to allow for making statements about the bias within these re-discretised intervals (too-long intervals would lead to mixing of underlying states; too-short intervals do not leave us with enough deviation from the original travel distances). We based this on the three following states: resting (no movement, state 1), slow velocities with uniformly distributed turning angles (state 2) and higher velocities with a higher likelihood for more straight movements (state 3). The parameters underlying these three states were fitted by a maximum-likelihood approach as implemented in the R package “moveHMM” (Michelot et al., 2016). We then compared the deviation from the original travel distance in metres per minute as introduced by re-discretisation of the original tracks on the 5 min grid, conditional on the reconstructed states by using the “Viterbi algorithm” on the basis of the hidden Markov model's results. Section S2 contains details on the states' parameterisations.

### Ethical approval

All research adhered to the legal requirements of the countries from which samples were obtained. The study was carried out in compliance with the principles of the American Society of Primatologists for the ethical treatment of non-human primates (https://www.asp.org/society/resolutions/EthicalTreatmentOfNonHumanPrimates.cfm). No animals were sacrificed or harmed for this study.

## 3 Results

### 3.1 Distances travelled

The database comprised 47 2 h tracks with 18 073 fixes, resulting in 18 026 legs. In 80.7 % of cases the GPS device was able to fix a position in less than 30 s. In only 0.6 % of cases it took between 45 and 60 s. Two-hour tracks lasted on average 2:00:06 h (n=47; SD = 5 s; min: 2:00:00 h; max: 2:00:18 h). Average leg time was 18.8 s (N = 18 026; SD = 11.0 s; min: 1 s; max: 60 s). Within each leg the average distance covered by the baboons was 5.1 m (N=18 026; SD = 5.5 m; range: 0–37.0 m).

**Figure 2:**
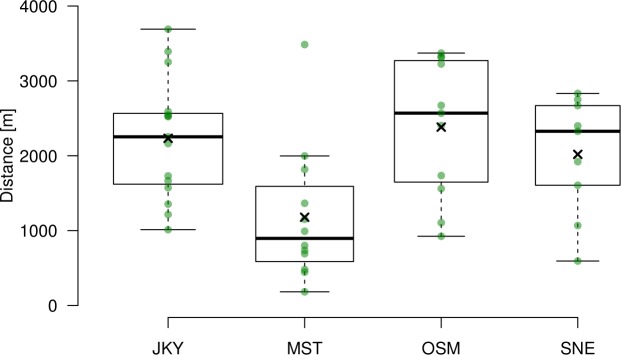
Inter- and intra-individual variation in distance travelled within 2 h. Median as thick solid horizontal line; 1st quartile $Q_{1}$ and 3rd quartile $Q_{3}$ as upper and lower box boundaries, respectively; whiskers calculated as upper whisker = $min(max\left(x\right),\;Q_{3}+1.5\cdot \mathrm{IQR})$ and lower whisker = $max(min\left(x\right),\;Q_{1}-1.5\cdot \mathrm{IQR})$, where IQR = $|Q_{3}-Q_{1}|$; and mean as black cross. Kruskal–Wallis test: H[3,N=47] = 10.924; p=0.012; $N_{\mathrm{JKY}}=15$; $N_{\mathrm{MST}}=12$; $N_{\mathrm{OSM}}=11$; $N_{\mathrm{SNE}}=9$.

The baboons travelled 1921 m within a 2 h track (median; range: 183–3691 m; N=47). However, among and within each subject there was considerable variation in speed of travelling and hence in distance covered within 2 h tracks (Fig. 2).

### 3.2 Deviation from true distance

**Figure 3:**
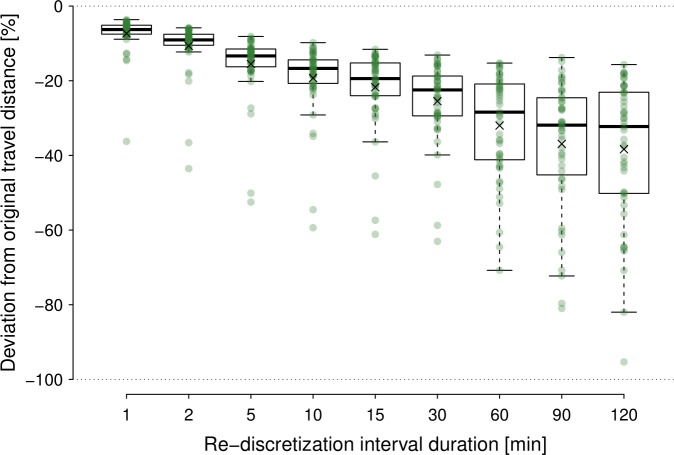
Deviation from original travel distance covered within 2 h (box plots illustrate the same descriptive statistics as described in the caption for Fig. 2), as revealed by applying different sampling intervals.

The deviation from the real distance covered within a 2 h track increased the longer the sampling interval was (Fig. 3). If we applied a 1 min sampling interval, we already underestimated the distance by 6.3 % on average (median). The deviation from the true distance increased to 32.3 % (median) if we used 2 h sampling intervals. Considerable variation in underestimating the distance could be observed, which can reach in the extreme case more than 80 % at 2 h sampling intervals.

**Figure 4:**
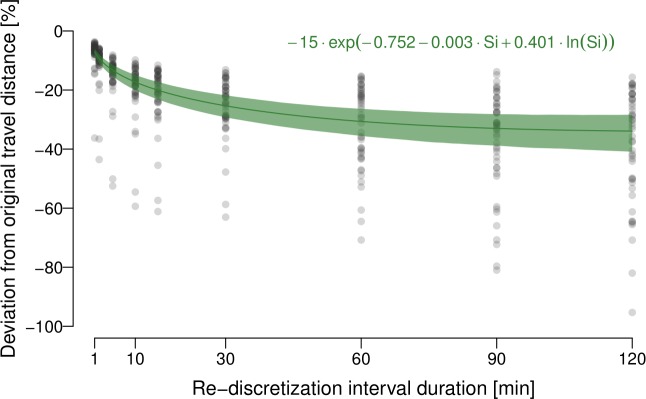
Expected deviation from the original travel distances (in %) conditional on re-discretisation interval duration (in minutes). The solid green line shows the estimated expectation (the functional form is described by the function as given on the top right of the figure); the green area shows a point-wise 99 % uncertainty interval for this estimated conditional expectation.

There is strong support that the expected deviation from the true distance follows an exponential function (Fig. 4), indicating that the relative error increase is larger at shorter sampling intervals, as can be seen in Fig. 4 by the estimated expected error levelling off with increasing re-discretisation interval duration.

**Figure 5:**
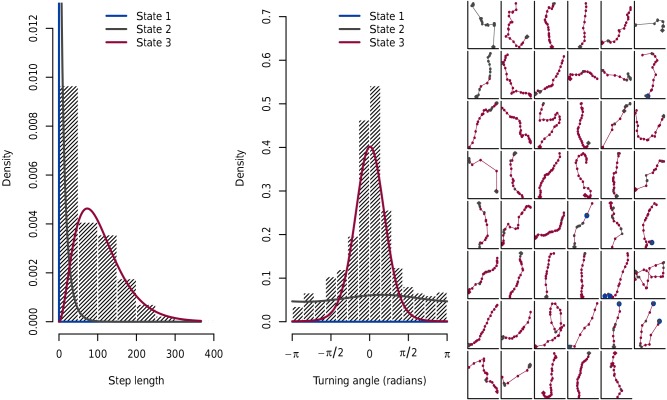
Results of the hidden Markov model estimation. Blue lines on the left and middle plots illustrate the densities for the “resting” state 1, dark grey lines show densities for state 2 (slow velocities, i.e. small step lengths per 5 min interval, approximately uniform distributed turning angles) and red lines illustrate the densities for state 3 (higher velocities and higher density for straight movements). The dashed histograms show the overall empirical distributions. The right figure shows the 47 tracks, coloured according to the states to which the Viterbi algorithm (based on the hidden Markov model results) categorised them (x and y axes are scaled such that each track can be seen in the maximal graphical solution; i.e. they are not equally scaled across the 47 plots).

### 3.3 Impact of states on deviation

**Figure 6:**
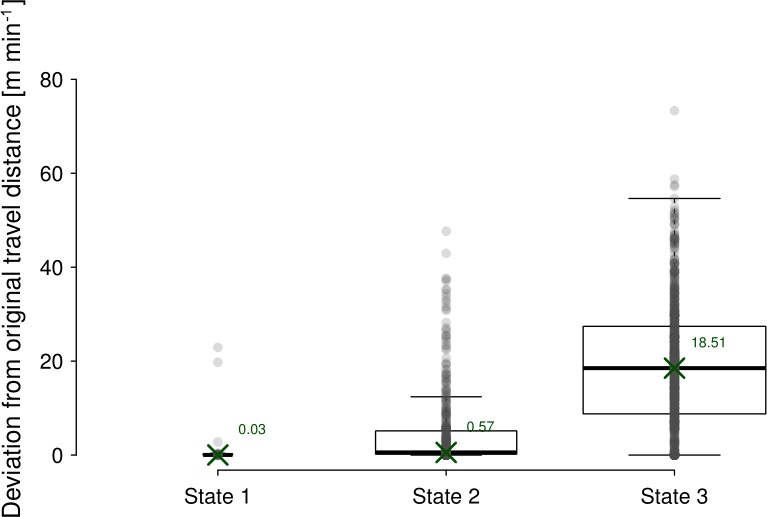
Deviation from original travel distance by the artificial 5 min sampling scheme, conditional on the states as estimated by the Viterbi algorithm applied on the hidden Markov model results shown in Fig. 5. Box plots illustrate the same descriptive statistics as described in the caption for Fig. 2, with values of the median conditional deviations given directly in the figure, and also illustrated by crosses.

The movement behaviour of the baboons – here categorised in three states: resting (no movement, state 1), slow velocities with uniformly distributed turning angles (state 2) and higher velocities with a higher density for more straight movements (state 3) – had a clear impact on the magnitude of error in estimating travel distances (Figs. 5 and 6). The deviation from true distance was of course smallest if the baboons did not move (stage 1) and largest if the baboons moved fast in a more or less straight direction. This appears counterintuitive at first glance but is explained by the much stronger consequences of even small turning angles at intervals with fast movement than at intervals with slow movement.

## 4 Discussion

Results of our analysis of the DTD of Guinea baboons met the general expectation: the higher the frequency of positions, the more trustworthy the movement paths (Nathan et al., 2008). The absolute average underestimate of DTDs was found to be less than 7 % for 1 fix min${}^{-1}$ and less than 35 % for 1 fix/120 min. However, it can reach up to a maximum of 30 and 90 % if respective sampling frequencies of 1 fix min${}^{-1}$ or 1 fix 120 min${}^{-1}$ are applied. The increase of deviation from true DTDs followed an exponential function, indicating that the average relative error increase is larger at shorter sampling intervals than at larger ones, which is in agreement with findings from Rowcliffe et al. (2012). For instance, whether, in the case of the Guinea baboons, we use a 90 or a 120 min sampling interval would not make a relevant difference in underestimating DTDs. At least for Guinea baboons we now have a reliable estimate of average underestimation of DTDs, which we can, for example, use as a correction when calculating absolute travel costs. We assume that similar magnitudes of underestimation of DTDs apply for other terrestrial primates, such as other baboon species.

The magnitude of error, however, is also dependent on the behaviour of the individuals under consideration. In the extreme, if an animal does not move over a long period, the true travelled distance is 0 and the deviation from the true value also becomes 0, irrespective of the length of the sampling interval. Similarly, it is very likely that the error remains small if an animal moves relatively slowly in a straight direction, whereas one can expect a large deviation if the animal moves quickly with a lot of meandering. In our study, we analysed the impact of movement behaviour exemplarily at a 5 min sampling interval. As expected, in state 1 (mainly resting) the deviation was minimal, whereas it increased minimally if the animal moved slowly (state 2) and increased more if it moved quickly (state 3). Since the spatial behaviour of baboons and other animals is often influenced by ecological condition (e.g. temporal and spatial distribution of food), one can expect season might affect step length and path tortuosity (Calenge et al., 2009; Owen-Smith et al., 2010).

Moreover, the time of day might play an additional role in shaping the characteristics of a travel path. In Chacma baboons (*Papio ursinus*), for example, Noser and Byrne (2010) found that their study group used two different strategies over the course of the day to exploit available fruit trees. In the early morning, the baboons showed a more goal-directed travel behaviour with linear travel routes and high movement speeds, whereas the baboons travelled more opportunistically (slower and less directly) during the rest of the day. DTDs can also vary among seasons if the spatial distribution of resources changes and forces individuals to adapt their DTDs (Hemingway and Bynum, 2005). If such temporal patterns in movement behaviour are significant in a species, the estimation of error in DTDs needs to be adapted to these patterns.

We were able to determine underestimations of DTDs in a terrestrial primate, the Guinea baboon, when applying different sampling intervals. The values of underestimation can be used as a corrective in estimations of absolute DTDs and travelling costs, which can make comparisons among different primate groups more reliable. Our analysis also showed, at least for terrestrial primates such as baboons, that there is no significant increase of underestimation beyond a sampling interval of 60 min (60, 90, 120 min). As shown in Fig. 4, this mainly results from a weaker increase in underestimation for larger interval durations, and less from an increase in estimation uncertainty. Such a priori knowledge on underestimations of DTDs is important to inform researchers conducting GPS remote telemetry studies. Based on analyses such as ours, researchers can choose the “appropriate” sampling intensity in order to optimise the trade-off between sampling density and battery longevity.

We think that the overall magnitude of error, as found in our baboon study, will provide an estimate transferable also to other terrestrial or semi-terrestrial primate species. However, if the respective species show largely deviating movement behaviour, the magnitude of error will most likely change.

## Supplement

10.5194/pb-4-143-2017-supplement The supplement related to this article is available online at: https://doi.org/10.5194/pb-4-143-2017-supplement.

## Data Availability

Data are provided as Supplement S3.
