# Supplementary material for: Estimation of baboon daily travel distances by means of point sampling – the magnitude of underestimation
Source: Primate Biol. 2017 Jul 10;4(2):143–51. doi: 10.5194/pb-4-143-2017 (PMC7041532; doi:10.5194/pb-4-143-2017)
Supplement: The supplement related to this article is available online at: https://doi.org/10.5194/pb-4-143-2017-supplement. [file pb-4-143-supplement.zip › pb-4-143-2017-supplement-title-page.pdf]

Supplement of Primate Biol., 4, 143–151, 2017
https://doi.org/10.5194/pb-4-143-2017-supplement
© Author(s) 2017. This work is distributed under
the Creative Commons Attribution 3.0 License.

Primate Biology
Open Access

Supplement of

Estimation of baboon daily travel distances by means of point sampling – the magnitude of underestimation

Holger Sennhenn-Reulen et al.

Correspondence to: Dietmar Zinner (dzinner@gwdg.de)

- pb-4-143-2017-supplement-title-page.pdf
- Sennhenn-Reulen_Supplement_S1_S2.docx
- Sennhenn-Reulen_Supplement_S3.xlsx

The copyright of individual parts of the supplement might differ from the CC BY 3.0 License.
