# Supplementary material for: Estimation of baboon daily travel distances by means of point sampling – the magnitude of underestimation
Source: Primate Biol. 2017 Jul 10;4(2):143–51. doi: 10.5194/pb-4-143-2017 (PMC7041532; doi:10.5194/pb-4-143-2017)
Supplement: The supplement related to this article is available online at: https://doi.org/10.5194/pb-4-143-2017-supplement. [file pb-4-143-supplement.zip › Sennhenn-Reulen_Supplement_S1_S2.docx]

**Supplement S1: Details to the Bayesian multilevel lognormal regression model**

The transformation of the response by inverse -15 was applied since there is good practical experience with the below prior assumption for a response that is divided by its standard deviation prior to estimation, and the negative sign to get to a strictly positive values that are suitable for the positive lognormal distribution. For the track index grouping factor $\gamma_{i}$ we made the usual assumption of normally distributed deviations from the group average, i.e. $\gamma_{i}\sim N(0, \sigma_{\gamma}^{2})$, with setting a weakly informative half student-t prior with scale parameter 10 on $\sigma_{\gamma}$. The Markov chain Monte Carlo algorithm was run for 3 chains, each with 2000 iterations and an initial burn-in of 1000 iterations.

Posterior mean and 95% credible interval (CI) for $\beta_{0},\beta_{1},\beta_{2}$:

|  | Posterior mean | Lower 95% CI bound | Upper CI bound |
| --- | --- | --- | --- |
| $\beta_{0}$ | -0.752 | -0.886 | -0.633 |
| $\beta_{1}$ | -0.003 | -0.004 | -0.002 |
| $\beta_{2}$ | 0.401 | 0.370 | 0.433 |

**Supplement S2: Hidden-Markov-Model Parameter Specifications**

We assume the underlying latent process – generating the travel behaviour – to be of an ordinally scaled three state-type process form: no movement (no adequate definition of turning angles, e.g. sitting; state 1), slow movement with a flat distribution of turning angles (e.g., searching for food in a small area; state 2), and fast movement with a high concentration of turning angles around 0 degrees (e.g. covering distances; state 3). We use the following starting parametrization for the distribution of segment lengths (in meters) for the maximum likelihood estimation algorithm provided by moveHMM (Michelot et al., 2016): $\mathrm{state}_{1}\sim\mathrm{Gamma}\left( 0.001,0.001 \right)$, $\mathrm{state}_{2}\sim\mathrm{Gamma}\left( 1,2 \right)$, and $\mathrm{state}_{3}\sim\mathrm{Gamma}\left( 7,7 \right)$. Since the artificially refined path with segment durations of 5 minutes contains segments with step lengths of 0 meters, we include the zero-mass parameters 0.999, 0.1, and 0.001 for the states 1, 2, and 3, to consider potential ‘zero-inflation’. For the turning-angle distribution between the path segments, we used the following start parametrizations for the von Mises distribution: turning-angle distribution between the path segments: $\mathrm{state}_{1}\sim\mathrm{vM}\left( 0,0.1 \right)$, $\mathrm{state}_{2}\sim\mathrm{vM}\left( 0,1 \right)$, and $\mathrm{state}_{3}\sim\mathrm{vM}\left( 0,6 \right)$. As maximum likelihood parameters, we get for the step lengths: $\mathrm{state}_{1}\sim\mathrm{Gamma}\left( 0.001,0.001 \right)$, $\mathrm{state}_{2}\sim\mathrm{Gamma}\left( 10.6,13.4 \right)$, and $\mathrm{state}_{3}\sim\mathrm{Gamma}\left( 108.9,62.6 \right)$, with zero-mass parameters 1, 0.07, and 0.004. For the turning angle distributions, we get: $\mathrm{state}_{1}\sim\mathrm{vM}\left( 0,9989 \right)$, $\mathrm{state}_{2}\sim\mathrm{vM}\left( 0.5,0.14 \right)$, and $\mathrm{state}_{3}\sim\mathrm{vM}\left( 0.01,2.95 \right)$. We see that the state 1 distribution is classifying segments with lengths of 0 meters almost completely, where the turning angle is actually not defined, leading to an ill-conditioned distribution of turning angles in this state. However, Figure 5 showed that the estimated segment length and turning angle distributions for states 2 and 3 seem to represent the empirical distributions quite nicely.
